# Update on Management Recommendations for Advanced Cutaneous Squamous Cell Carcinoma

**DOI:** 10.3390/cancers14030629

**Published:** 2022-01-27

**Authors:** Jesús García-Foncillas, Antonio Tejera-Vaquerizo, Onofre Sanmartín, Federico Rojo, Javier Mestre, Salvador Martín, Ignacio Azinovic, Ricard Mesía

**Affiliations:** 1 Departamento de Oncología, Hospital Universitario Fundación Jiménez Díaz, 28040 Madrid, Spain; FRojo@fjd.es (F.R.); Ignacio.azinovic@quironsalud.es (I.A.); 2 Department of Medicine, Faculty of Medicine, Universidad Autónoma de Madrid, 28040 Madrid, Spain; 3 Instituto Dermatológico GlobalDerm, Palma del Río, 14700 Cordoba, Spain; antoniotejera@aedv.es; 4 Unidad de Oncología Cutánea, Hospital San Juan de Dios, 14012 Cordoba, Spain; 5 Instituto Valenciano de Oncología, 46009 Valencia, Spain; osanmartinj@gmail.com; 6 Hospital Universitario Miguel Servet, 50009 Zaragoza, Spain; javiermestre@yahoo.com; 7 Clínica Universitaria de Navarra, 28027 Madrid, Spain; smalgarra@unav.es; 8 B-ARGO Group, Medical Oncology Department, Institut Català d’Oncologia (ICO), Badalona, 08908 Barcelona, Spain; rmesia@iconcologia.net

**Keywords:** cutaneous squamous cell carcinoma, prognosis, multidisciplinary management, surgery, systemic therapy

## Abstract

**Simple Summary:**

Cutaneous squamous cell carcinoma (cSCC) is the second most common form of skin cancer, which predominantly occurs on the head and neck. Early detection and treatment of primary tumours is crucial to limit progression and local invasion of deep tissues. While high-risk markers of poor prognosis have been identified, factors predicting regional control or survival remain uncertain. Therefore, diagnosis and management of cSCC should be performed individually, considering patient’s clinicopathological profile and the best available treatment options. Surgical excision, radiotherapy, and/or systemic treatments can be selected depending on patient’s status and tumour stage. Considering that a more comprehensive assessment will be provided by a multidisciplinary team, we aimed to generate a practical document that may assist oncologists and dermatologists on the prognosis, diagnosis, management, and follow-up of patients with advanced cSCC.

**Abstract:**

Cutaneous squamous cell carcinoma (cSCC) is the second most common form of skin cancer, the incidence of which has risen over the last years. Although cSCC rarely metastasizes, early detection and treatment of primary tumours are critical to limit progression and local invasion. Several prognostic factors related to patients’ clinicopathologic profile and tumour features have been identified as high-risk markers and included in the stratification scales, but their association with regional control or survival is uncertain. Therefore, decision-making on the diagnosis and management of cSCC should be made based on each individual patient’s characteristics. Recent advances in non-invasive imaging techniques and molecular testing have enhanced clinical diagnostic accuracy. Surgical excision is the mainstay of local treatment, whereas radiotherapy (RT) is recommended for patients with inoperable disease or in specific circumstances. Novel systemic treatments including immunotherapies and targeted therapies have changed the therapeutic landscape for cSCC. The anti-PD-1 agent cemiplimab is currently the only FDA/EMA-approved first-line therapy for patients with locally advanced or metastatic cSCC who are not candidates for curative surgery or RT. Given the likelihood of recurrence and the increased risk of developing multiple cSCC, close follow-up should be performed during the first years of treatment and continued long-term surveillance is warranted.

## 1. Introduction

Cutaneous squamous cell carcinoma (cSCC) is one of the most relevant non-melanoma skin cancers (NMSC), along with basal cell carcinoma (BCC). With more than 700,000 cases diagnosed each year in the U.S. [1], the likelihood of patients developing at least a second cSCC is 13% [2]. In Spain, cSCC is the second cause of skin cancer-related mortality, with an estimated incidence of 40 cases per 100,000 person-years [3]. It predominantly occurs on the head and neck (cSCCHN). Sun exposure, particularly chronic UV exposure, is the most relevant risk factor for cSCC [4]. Artificial ultraviolet radiation, including PUVA therapy with a load of more than 350 sessions, has also been associated with a higher incidence of cSCC [5]. Immunosuppression, especially in the context of solid organ transplants and patients with chronic lymphocytic leukaemia, is associated with a higher frequency among younger patients and more aggressive carcinomas [6].

Diagnosis of advanced cSCC should involve experts from different specialties, including dermatologists, anatomical pathologists, surgeons, radiation oncologists, and medical oncologists, thereby enabling a more comprehensive patient assessment. Likewise, decision-making for patient management and follow-up should be guided by this multidisciplinary approach, selecting the best available therapeutic options from an individualized perspective [7,8]. In recent years, increased knowledge of molecular biology and the implementation of novel techniques for surgery and radiotherapy (RT) have led to new treatment lines which have significantly improved the chance of local control and survival. Moreover, a broader therapeutic arsenal beyond surgery and RT is currently available that includes novel systemic treatments, such as new chemotherapeutic agents, targeted therapies, or immunotherapies [8,9].

This review was prompted by several considerations. First, while the recent update of tumour classification system has improved its prognostic value, the most relevant factors predicting patient outcomes remain unclear. Second, standardized protocols are required to ensure the consistency of clinical and pathological reports, thus allowing the appropriate assessment of clinical risk from a multidisciplinary approach. Finally, a practical document that includes the characteristics, usefulness, and role of diagnostic tests and available treatments may assist both oncologists and dermatologists. To further support the evidence herein addressed and its applicability in clinical practice, a questionnaire was conducted among a multidisciplinary panel of specialists with the aim of providing consensus recommendations on the prognosis, diagnosis, treatment, and follow-up of cSCC (References [10,11,12] are cited in the Supplementary Material).

## 2. Tumour Staging and Prognostic Factors

Tumour staging of cSCC arising from the head and neck region is based on the American Joint Committee on Cancer (AJCC) classification, 8th ed. [13], which additionally considers tumour thickness [14], perineural infiltration diameter, and tumour invasion depth to classify primary tumours [15] (Table 1). Alternatively, the Brigham and Women’s Hospital (BWH) classification has also been proposed as a simpler and more intuitive classification system for localized stage disease. The BWH system may be a better predictor of regional nodal relapse or disease-related mortality than AJCC 8th ed. [16], although no differences have been observed for immunosuppressed patients [17]. In a recent systematic review, the BWH and AJCC systems similarly predicted the presence of metastasis after selective sentinel node biopsy [18].

Tumour staging according to the TNM classification, which is applicable for cSCC from all regions, is shown in Table 2. The N category referring to regional metastatic affectation is based on a scheme shared with other head and neck tumours. It should be noted that the AJCC classification system is only valid for these tumours. Other prognostic factors not included in this classification, such as degree of differentiation, growth rate [21], and the presence of budding, have also been described [22].

## 3. Molecular Pathology and Emerging Biomarkers

**Figure 1:**
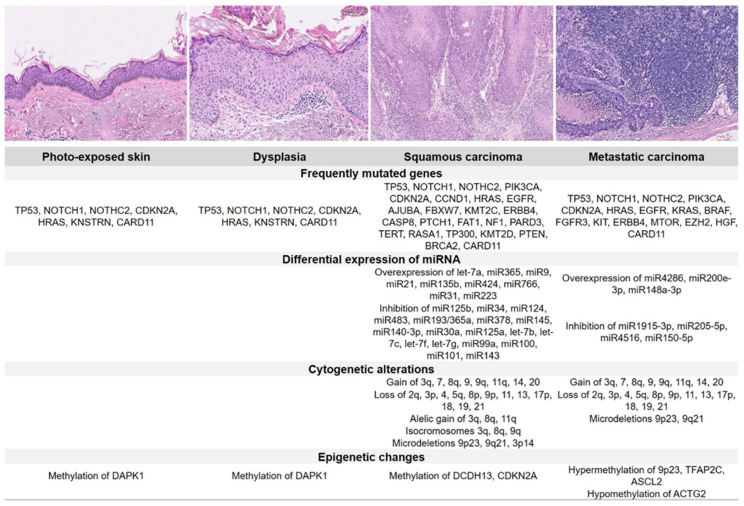
Histology and molecular pathology of photo-exposed skin, dysplasia, squamous carcinoma, and metastatic carcinoma.

cSCC presents a higher number of mutations than other tumours, i.e., up to 5 times that of lung cancer or 4 times that of melanoma [23]. By accumulating these mutations, an area of skin can—usually in response to UV light damage—progress through increased levels of dysplasia to become a cSCC, and in fact, areas of photo-exposed skin share many of the same carcinoma features [24]. Although there is a large range of tumour mutational burden (TMB) in cSCC, the median TMB is the highest compared with other tumour types. Exome sequencing in the cSCC has revealed approximately 1300 nucleotide somatic variations per exome. This observation has led to the hypothesis that constant damage to basal keratinocytes by UV radiation may be responsible for multiple cSCC mutational events [25] (Figure 1).

The most frequent mutations in cSCC are associated with ultraviolet radiation and affect TP53 and NOTCH signalling pathway genes. While mutations in the TP53 family decrease the expression of other tumour suppressors, such as IFR6 [26], modifications in NOTCH signalling may have an oncogenic or suppressor role depending on the cellular microenvironment [27]. Activating mutations on genes of the RAS family, mainly HRAS, have been identified in up to 9% of SCC [25], with increasing incidence in up to 60% of tumours developed in patients treated with BRAF/MEK inhibitors [28]. CDKN2A, which is mutated in 31% of primary and metastatic SCC, is associated with tumour aggressiveness [29] and epigenetic changes due to hypermethylation of the CpG islands of the FRZB, TFAP2C, ASCL2 [30], and KMT2C [23] genes. Less frequently, alterations in STAT3, PIK3CA, KIT, RIPK4, and RAS1 genes have been also described [23,31]. Evidence also suggests that phenotypic changes caused by tumour–stromal interaction and the secretion of VEGF-C by tumour-associated fibroblasts might be involved in the cSCC metastatic process [32,33].

## 4. Diagnosis

### 4.1. Dermatology

In recent years, optical non-invasive diagnostic techniques have been increasingly applied in clinical practice to improve diagnostic accuracy and to characterise the tumour in vivo before surgery or biopsy [9,34]. Dermoscopy can be used to identify the main characteristics of cSCC, such as clustered vascular pattern, glomerular vessels, and hyperkeratosis. Several additional features can be observed using reflectance confocal microscopy, such as atypical honeycomb or disarranged pattern of the spinous-granular layer of the epidermis, round nucleated bright cells in the epidermis, and round vessels in the dermis [35].

**Figure 2:**
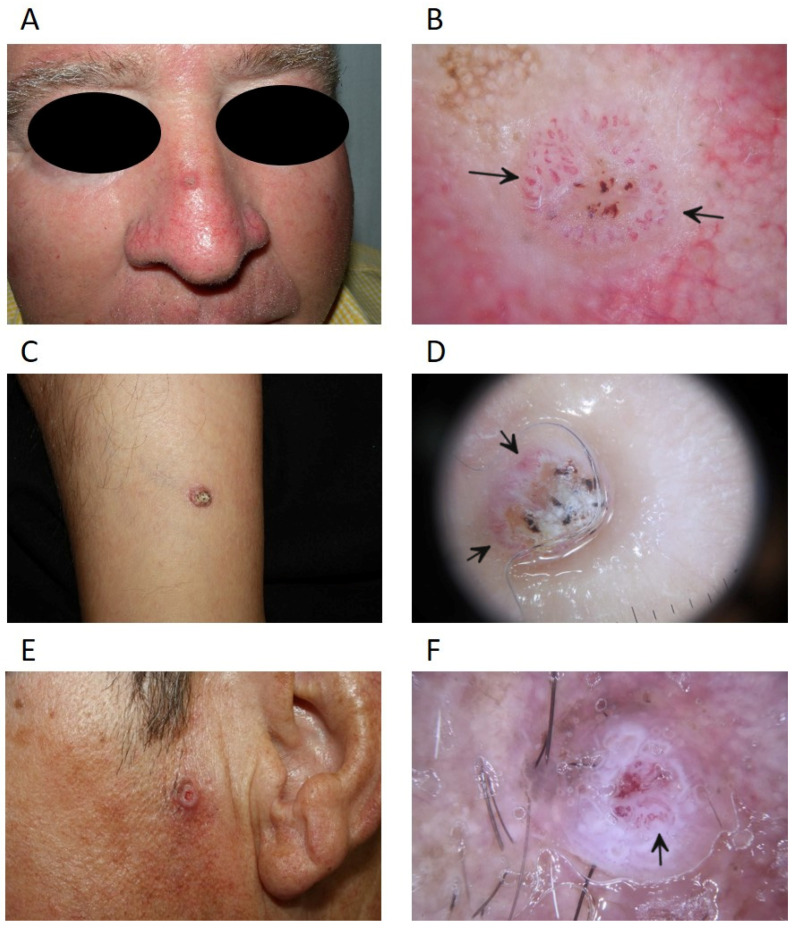
Dermoscopy of cutaneous squamous cell carcinoma. (**A**) Wart-like tumour lesion on the dorsum of the nose; (**B**) dermoscopy with polarised light showing a predominantly vascular pattern with serpentine, hairpin and irregular vessels (arrows), central ulceration and blood staining; (**C**) crateriform keratinising tumour lesion; (**D**) polarised light dermoscopy with central whitish crust and presence of irregular and comma-shaped vessels (arrows) in the periphery; (**E**) crateriform tumour lesion; (**F**) polarised light dermoscopy showing white unstructured areas with irregular groups of white perifollicular circles, central vascular pattern with hairpin and irregular vessels (arrows).

While dermoscopy is performed more often in pigmented than in non-pigmented lesions, it is also useful in the differential diagnosis of equivocal cases. For instance, the identification of glomerular-like, clustered or hairpin vessels, and the scale and alignment of dots and vessels can be useful in particular scenarios, such as in minimally invasive cSCC or in pigmented forms [19]. In situ, cSCC frequently presents clinically as an erythematous scaly patch or slightly elevated plaque, whereas invasive cSCC is usually ulcerated and can be patchy, papulonodular, papillomatous, or exophytic [9]. Progression of in situ cSCC to microinvasive cSCC can be detected on dermoscopy examination with the appearance of a thicker lesion, hairpin and/or linear-irregular vessels, and a keratotic centre and/or ulceration (Figure 2). In invasive cSCC, a vertical growth phase reflecting dermal invasion is typically characterized by an increased number of polymorphic vessels, such as linear irregular, hairpin, and grouped glomerular/dotted vessels over a whitish background with a central mass of keratin or ulceration [9,35,36].

### 4.2. Histopathology

The histological diagnosis of cSCC is made using haematoxylin–eosin staining, although in cases of uncertain diagnosis, especially in non-keratinizing tumours, additional immunohistochemical studies for cytokeratins or stratified epithelia may be necessary. The morphological image shows bundles of atypical keratinocytes originating in the epidermis and infiltrating the dermis. Morphological features of differentiation can include the formation of corneal pearls, parakeratosis, and dyskeratosis. cSCC ranges from well-differentiated tumours to poorly differentiated neoplasms that show pleomorphic nuclei with a high degree of atypia, frequent mitosis, and very little keratin formation, if any [37].

Well-differentiated histological subtypes with low metastatic potential have been described, including keratoacanthoma and verrucous and fusocellular carcinoma, as well as other aggressive subtypes, such as acantholytic squamous, desmoplastic, and adenosquamous carcinomas, that have a risk of metastasis of 16.1% (95% CI 6.6–39.5) [14]. Other relevant morphological prognostic factors include the degree of dermal infiltration, tumour diameter (greater than 2.0 cm), the presence of perineural (especially in nerves greater than 0.1 mm) and lymphovascular invasion, and resection margins [38].

### 4.3. Medical Imaging

Radiological investigations are indicated when extensive disease is suspected, to determine bone or soft tissue involvement, invasion of surrounding areas, and the development of metastasis [7]. Main characteristics of imaging modalities used in cSCC are summarized in Table 3.

Computed tomography (CT) scans evaluate cartilage and bone involvement, and three-dimensional imaging is very useful in planning the surgical approach and subsequent reconstruction. Magnetic resonance imaging (MRI) is preferred to assess the involvement of deep soft tissue and structures such as bone, parotid glands, and major nerves [19,40]. Ultrasound (US) is a very sensitive method for identifying lymph node metastases and serves to guide the radiologist during needle biopsy [7,19]. Positron emission tomography (PET) is useful for detecting metastases in sites where other studies are inconclusive, as in the case of fibrosis, necrosis, and previously radiated tissues. However, it should be noted that the presence of infectious and inflammatory processes may lead to false positives [40]. The use of combined PET-CT increases CT sensitivity [40], thus permitting the accurate detection of metastasis in distant organs [19].

### 4.4. Assessment of Comorbidities

In NMSC patients, three comorbidity assessment tools are frequently used in clinical trials and could be considered in clinical practice: the Charlson Comorbidity Index (CCI), the American Society of Anesthesiologists (ASA) risk classification system, and the Adult Comorbidity Evaluation-27 (ACE-27) [41]. In a systematic review of 22 studies, the CCI was used most often to assess comorbidities (82% of cases), probably because of its extensive use among skin cancer patients and validation in other cancer populations [41]. While higher CCI scores were significantly associated with age > 80 years in patients with head and neck tumours [42], correlation between CCI and life expectancy in nonagenarians undergoing Mohs micrographic surgery is still unclear [43,44].

The ASA risk classification has been traditionally used as a predictor of risk in the preoperative screening of patients undergoing general anaesthesia [45]. Although it was not formally developed as a comorbidity index, its widespread use enables data retrieval from medical records of surgical skin cancer patients [41]. The ACE-27, which is a modification of the Kaplan–Feinstein Index, performs better than a standard medical interview in the identification of comorbidities among skin cancer patients [46]. Since it includes more comorbid conditions and enables further grading than the CCI score, the ACE-27 could potentially predict the prognosis of patients more accurately. However, larger studies are warranted [41].

## 5. Risk Stratification

Current prognostic factors for recurrence, which help determine the role of definitive or adjuvant treatments, are based on clinicopathologic features described in single-centre or large-scale clinical studies or consensus meetings. These factors have been established taking into account local staging, location, depth, and pathological features. The NCCN guidelines establish low- and high-risk features for cSCC (Table 4) [7]. High-risk features are observed in approximately 5% of all NMSC and include locally advanced disease (stages T3-4), nodal involvement, perineural invasion (PNI), local and regional recurrence, or immunosuppression [47].

PNI is a well-known high-risk factor for local or distant relapse that affects 5% of patients, mostly in cSCC [48]. In a systematic review, patients with clinical PNI (CPNI) showed a significantly increased risk of local recurrence (37% vs. 17%) and disease-specific death (27% vs. 6%) compared with patients with incidental PNI (IPNI), whereas nodal and distant metastasis were similar regardless of PNI classification. In addition, CPNI had significantly poorer mean 5-year recurrence-free survival (61% vs. 76%) and disease-specific survival (70% vs. 88%) than IPNI patients [49].

## 6. Treatment

### 6.1. Role of Surgery in Primary Tumours

**Figure 3:**
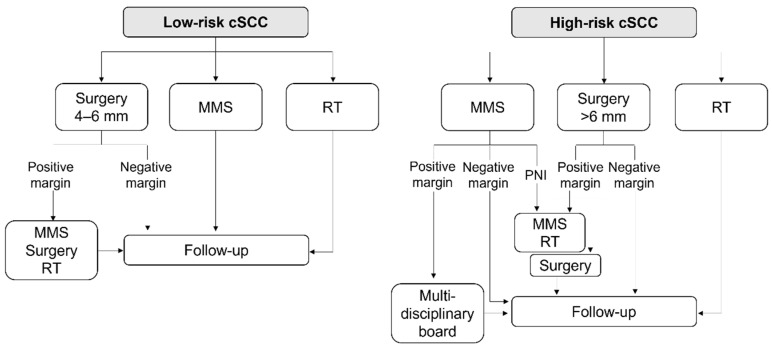
Therapeutic algorithm for low- and high-risk SCC. cSCC, cutaneous squamous cell carcinoma; MMS, Mohs micrographic surgery; PNI, perineural invasion; RT, radiotherapy.

Whenever possible, and taking into account the patient’s status, surgical excision of the tumour is the first-line treatment for cSCC patients, regardless of age and anatomical location [8]. The choice among available modalities, which include conventional surgery and micrographic-controlled surgery, should be based on the patient’s risk factors for poor prognosis (Figure 3).

Mohs micrographic surgery (MMS) may be the preferred technique for high-risk patients based on the high cure rates and low recurrence rates reported in retrospective analyses of primary and recurrent cSCC [7]. Alternatively, patients may undergo conventional surgery and intraoperative biopsies. Wide local excision may be more appropriate for large and invasive lesions, as it allows for complete assessment of peripheral and deep margins. To achieve histologically clean margins after surgical excision, the width of the margins should be adapted to the risk of cSCC extension and recurrence. For low-risk lesions, clinical practice guidelines recommend performing an excision with 4–6 mm margins [8]. However, larger margins are needed for larger tumours and when high-risk features, such as perineural or lymphatic invasion, are present. While margins > 6 mm would be required for lesions with a diameter > 1 cm [7], the most appropriate width should be determined by individual risk assessment [8]. From the oncological perspective, a surgical defect should be restored using techniques that do not mobilize surrounding tissues, such as direct closure or grafting [7], while reconstruction with skin flaps may be only advised after ensuring that the tumour has been completely removed.

Particular scenarios have been also described, such as cSCC that develop in association with scars or chronic wounds (Marjolin’s ulcer) or previously radiated skin, those that invade deep structures, such as bone, parotid or nerve trunks, and those located in the labial vermilion and the ear [7]. In these cases, amputation of the affected limb, parotidectomy, and prophylactic lymph node drainage may be necessary.

### 6.2. Locoregional Assessment and Sentinel Node Biopsy

Sentinel lymph node biopsy (SLNB) in patients with cSCC is aimed at the early detection and management of occult nodal metastasis. According to two systematic reviews that included 16 and 23 studies, the positive SLNB rate among cSCC patients ranged between 14% and 8%, respectively [50,51]. A meta-analysis of 19 studies identified microscopic sentinel node involvement in 12.3% of patients who had a tumour diameter greater than 2 cm in all cases. Using the AJCC criteria, a higher risk of positive SLNB was associated with advanced tumour stages, reaching 29.4% in T2b and 50.0% in T3 lesions [52].

The utility of SLNB in detecting cSCC micrometastases not identified by non-invasive examination methods (i.e., instrumental tests) has been discussed. Notably, the rate of micrometastases increased from 3.4% in the overall population to 6.5% in the high-risk group; this rate directly correlated with the depth and diameter of the tumour [53]. In a retrospective analysis of cSCC patients who underwent SLNB and did not present micrometastasis, no relapse event nor local/distant metastases were reported during a mean follow-up of 27.5 months [54].

Nonetheless, given the lack of high-level evidence supporting a real prognostic impact and a well-defined profile of patients who could benefit the most, SLNB is not currently recommended in patients with invasive cSCC outside of a clinical trial setting [19].

### 6.3. Local Treatment

Surgical excision should be always considered as first-line treatment option for patients with either high- or low-risk cSCC. Nonetheless, based on the risk stratification, local approaches may be considered in patients for whom surgery is not feasible. For low-risk cSCC, several non-surgical treatment modalities are currently available to treat the tumour and field cancerization. Photodynamic therapy consists of a 2-step method that involves the topical application of a photosensitizer, such as 5-aminolevulinic acid or methyl aminolevulinate, followed by an incubation period with light irradiation. Alternatively, a topical therapy with imiquimod up to 5% or 5-fluorouracil may be also applied [9].

### 6.4. Role of Radiotherapy

RT may be used in first line in patients unable to undergo surgical resection, such as cosmetic or frail patients, to avoid significantly disfiguring surgery or orbital exenteration or in patients too frail to undergo general anaesthesia. A recent meta-analysis involving 21,000 patients showed comparable, extremely low one-year recurrence rates for both surgery and RT: 0.8%, 0.2%, 2%, and 0% for surgical excision, MMS, external beam irradiation, and brachytherapy (BT), respectively, and excellent cosmesis [55]. Different RT techniques, such as external beam RT, BT or electronic low energy sources (ELS), and schedules (i.e., normal fractionation or hypofractionation) can be selected depending on the expertise of the hospital. More recently, newer technologies such as volumetric arc therapy (VMAT) have proven useful in the treatment of scalp lesions or extensive field cancerization in trunk or extremities unamenable to other radiation modalities [56].

The role of RT in NMSC, mainly cSCC, has been defined in a recently published clinical practice guideline, following the ASTRO recommendation grading system [57]. Definitive RT is: (a) advised for patients who cannot undergo or who decline surgery, (b) conditionally recommended in anatomic locations where surgery can compromise function or cosmesis, and (c) not recommended (conditionally) in genetic diseases predisposing to higher radiosensitivity (ataxia-telangiectasia, Gorlin syndrome, Li–Fraumeni syndrome). Definitive RT is only recommended for inoperable patients, whereas elective node irradiation is conditionally recommended in lesions (thickness > 6 mm) in which there is an overlap of the primary tumour with the adjacent nodal basin.

Adjuvant RT is strongly recommended in NMSC patients with gross PNI as well as for cSCC patients with any of the following characteristics: (a) close or positive margins not amenable to re-resection, (b) relapse after prior margin-free surgery, (c) stage T3–T4, (d) desmoplastic reaction or lymphocyte infiltration in chronic immunosuppression. In patients with regional node metastasis, adjuvant RT after therapeutic lymphadenectomy is strongly recommended except for single, <3 cm nodes without extracapsular invasion. The addition of concurrent carboplatin chemotherapy to postoperative RT has not been shown to improve locoregional control in patients with high-risk cSCCHN [47].

For adjuvant RT, conventional doses of 60–66 Gy (2 Gy/day) are the standard, most frequently used fractionation. In case of elective RT, doses of 50–54 Gy are recommended [57]. Hypofractionation (HF) is usually delivered for brachytherapy or ELS, most often at doses of 50 Gy (20 fractions, 4 weeks) or 45 Gy (15 fractions, 3 weeks). Alternatively, doses of 30–40 Gy (5–7 Gy fractions, 1–3 fractions per week) result in excellent local control rates and acceptable toxicity. HF is most advantageous in smaller lesions, frail patients, elderly patients, or anyone with problems coming into the clinic on a daily visit [58,59]. Zaorsky et al. recommend schedules of 50 Gy/15 fractions, 36.75 Gy/7 fractions, or 35 Gy/5 fractions, as cosmesis outcomes are “good” in 80% of patients, depending on frailty [59]. Suggested treatments with RT using external beam irradiation are shown in Table 5.

### 6.5. Systemic Treatment

#### 6.5.1. Chemotherapy

Systemic treatment is administered to patients with cSCC who have progressed locally and/or with metastases on previous local treatments. Although no chemotherapeutic agents have been specifically approved for cSCCHN, platinum, 5-fluorouracil, anthracycline, or bleomycin alone or in combination are frequently used [60,61]. Moreover, the combined administration of cisplatin, interferon alfa, and 13-cis-retinoic acid has shown to be clinically active in patients with advanced cSCC, resulting in 34% overall and 17% complete response (CR) rates, with a median survival of 14.6 months [62].

#### 6.5.2. Targeted Therapy

Epidermal growth factor receptor (EGFR) expression is present in 90% of cSCC tumours, with overexpression in up to 35% [63]. Four phase II trials tested EGFR tyrosine kinase inhibitors (TKIs), erlotinib [64] and gefitinib [65], and monoclonal antibodies cetuximab [66] or panitumumab [67] in the recurrent/metastatic setting (Table 6). Treatment with TKIs resulted in lower response rates without CR compared with antibodies but with similar disease control and duration of response. Likewise, survival outcomes did not differ, with a median progression-free survival (PFS) of 4–8 months or overall survival (OS) of 8–13 months. The largest cohort reporting clinical outcomes of cetuximab alone in unselected patients with unresectable or metastatic tumours confirmed a high disease control rate of 87% (53% response rate) [68].

In contrast with panitumumab or TKIs, cetuximab demonstrated efficacy in cSCCHN [69,70], so it has been widely used in this setting in monotherapy or combined with chemotherapy [71]. Notably, the administration of cetuximab with chemotherapy and/or anti-EGFR has been associated with significant toxicity, mainly cutaneous. Comorbidities should be evaluated, and geriatric assessment should be performed in elderly patients with cSCC to identify the most effective and tolerable therapy for each patient.

#### 6.5.3. Immunotherapy

Immune checkpoint inhibitors (ICIs) have drastically improved survival outcomes in patients with advanced melanoma, with a considerable proportion of long-term survivors [72]. As tumours with a high mutational burden are known to be more responsive to ICIs and this mutational burden is up to 4 times greater in cSCC than melanoma [33], clinical trials were conducted in patients with cSCC [73]. Currently, anti-programmed cell death-1 (anti-PD-1) antibodies constitute first-line systemic treatment for metastatic or locally advanced cSCC in which curative surgery or RT are not feasible [8]. All systemic treatments are off-label, except for the anti-PD-1 agent cemiplimab, approved by the FDA/EMA in patients with locally advanced or metastatic cSCC who are not candidates for curative surgery or curative radiation [8,74,75].

Cemiplimab is a high-affinity, highly potent human immunoglobulin G4 anti-PD-1 receptor monoclonal antibody [76] that has demonstrated efficacy (overall response rate (ORR) 46.1%) and long-term durable response with very effective disease control (disease control rate: 72.5%) in patients with advanced and metastatic cSCC [77,78]. With a median age of 72 (38–96) years, 67.9% had cSCCHN, 59.6% had metastatic disease, and 40.4% locally advanced cSCC. Up to 66.3% of patients received cemiplimab as first-line treatment, and median observed time to response was 2.1 (1.9–3.7) months. Overall, the CR rate was 16.1% and median time to CR was 11.2 months [78]. Patients had deepening responses over time as evidenced by increasing CR rates [77,78,79]. Estimated median PFS was 18.4 months (95% CI: 10.3–24.3), while median DOR and OS had not been reached after 15.7 months follow-up. In responding patients, the estimated proportion of patients with ongoing response was 87.8% (95% CI: 78.5–93.3) at 12 months and 69.4% (95% CI: 55.6–79.6) at 24 months [78]. Advanced cSCC patients treated with cemiplimab experienced clinically meaningful improvements in global health status/health-related quality of life, and pain and functional status, and they maintained a low symptom burden [80]. Recently, data from real-world practice have confirmed a similar benefit of cemiplimab to that observed in clinical trials in a cohort of 240 patients with advanced cSCC [81].

Along with cemiplimab, other anti-PD-1 agents are under evaluation in cSCC (Table 7). Pembrolizumab is being tested in a phase II study of patients with recurrent/metastatic or locally advanced unresectable cSCC (NCT03284424). Results of a first interim analysis showed effective antitumour activity with 34.3% ORR and clinically meaningful, durable responses [82]. Pembrolizumab is also being evaluated in patients with locally advanced cSCC after surgery and radiation (NCT03833167). The efficacy of nivolumab monotherapy in patients with locally advanced/metastatic cSCC is being investigated in phase II studies (NCT04204837, NCT03834233).

## 7. Immunosuppressed Patients

Immunosuppressed solid organ transplant recipients (SOTRs) present a 65- to 250-fold greater incidence of cSCC than the general population, more aggressive progression, and a higher risk of metastasis and death [83,84]. Moreover, certain transplant recipients are more likely to develop cSCC than others; an increased risk has been reported for patients who undergo heart transplant compared with kidney or liver recipients [85]. In this respect, the type, intensity, and duration of the immunosuppressive regimen seem related to the development of cSCC in SOTRs. A lower incidence of cSCC has been reported in patients who receive sirolimus compared with those treated with calcineurin inhibitors [83].

Immune status has been identified as a strong prognostic factor for disease outcomes in immunosuppressed patients. A multi-institutional study including patients with cSCCHN reported significantly lower 2-year locoregional recurrence-free survival and PFS in immunocompromised patients compared with immunocompetent patients. Moreover, immunosuppressed status, recurrent disease, poor differentiation, and PNI were significantly associated with locoregional recurrence in this population [86].

A close clinical follow-up, every 3–6 months lifelong, and tailored immunosuppressive treatments, with adjustment or reduction of maintenance post-transplant therapy, may be necessary to reduce the risk of new cSCC [8,19]. Dermatologists, transplant physicians, and patients must collaborate to ensure adherence to dermatologic surveillance recommendations and must monitor suspicious lesions, thereby reducing the risk of cSCC in SOTRs [87,88].

It should be noted that although immunosuppression of cSCC patients is often described in the setting of SOTRs, other conditions should not be disregarded. As such, patients immunosuppressed due to human immunodeficiency virus (HIV) infection, allogenic bone marrow transplant, or autoimmune diseases should be carefully monitored.

## 8. Follow-Up and Monitoring

Patients with cSCC should be closely followed up for recurrences and development of new keratinocyte cancers and melanoma, particularly if they have a history of cSCC [8]. Evidence from cohort studies and registries shows that patients with at least one cSCC are at risk for developing additional cSCC and other skin cancers [20]. Therefore, it is recommended that follow-up for all patients should include regular clinical assessment, including inspection and palpation of the excision site, the in-transit route, and the regional lymph nodes [7,8]. Patients who have had cSCC should receive counselling regarding the risk for new primary skin cancers, such as BCC, cSCC, and melanoma, and the benefits of in-office and self-screening for early detection should be made clear [7,20].

The frequency of visits should be adjusted individually, depending on patient-specific underlying risk characteristics for cSCC [7,8]. The recently updated interdisciplinary European guidelines for the management of invasive cSCC recommend scheduling follow-up examination based on low- or high-risk common primary, advanced, or regional disease and the immunosuppressive setting [8]. While patients with low-risk cSCC may be assessed every 6–12 months for 5 years, high-risk patients should be examined in the clinic every 3–6 months for 2 years (including lymph node US), every 6–12 months from year 3–5, and annually thereafter. Closer follow-up is advised in patients with locally advanced/metastatic cSCC or after surgery for locoregional metastases, including clinical and US evaluation every 3 months for 5 years and then once or twice per year [8]. Similarly, the National Comprehensive Cancer Network (NCCN) guidelines for cSCC recommend that the frequency of patients’ follow-up should depend on whether the tumour has spread to lymph nodes. Local cSCC should be monitored at least every 3–12 months during the first 2 years, every 6–12 months for another 3 years, and then annually for life. For regional cSCC, a more frequent history and clinical examination is advised: every 1–3 months for 1 year, every 2–4 months for 1 year, every 4–6 months for another 3 years, and then every 6–12 months for life [7].

Imaging tests (CT, MRI, or PET-CT) for non-palpable regional lymph nodes should also be performed in patients with regional cSCC [7] or locally advanced or metastatic disease [8]. The NCCN guidelines establish that the frequency of imaging scans should be tailored to individual clinical factors [7], whereas the European guidelines recommend examination every 3–6 months in the first 3 years and then based on individual symptoms and stage [8].

It should be noted that increased surveillance may be required for cSCC patients at very high risk of other primary tumours and recurrence, such as immunosuppressed patients or individuals with haematological comorbidities, genetic predisposition, or previous history of cSCC [7,8]. Their follow-up schedule, including clinical and imaging evaluation, should be modified depending on the characteristics of individual primary tumours (e.g., number and frequency of development) [8].

## 9. Summary and Conclusions

Tumour staging, management decisions, and monitoring of the cSCC are continuously improving thanks to the development of novel diagnostic tools and therapeutic agents. Multidisciplinary teams may ensure that the most appropriate strategy, tailored to the patient’s individual profile, is undertaken from the initial diagnosis to follow-up. Patient-specific characteristics for risk including comorbidities, clinical parameters, histopathology, and molecular biomarkers should be considered to provide an accurate diagnosis. While surgery remains the cornerstone of treatment and RT is often used in the management of cSCC, ICIs and targeted therapies have changed the therapeutic landscape, showing improved outcomes in patients with locally advanced or metastatic disease. Considering the likelihood of recurrence and the increased risk of developing multiple cSCC, particularly in immunosuppressed patients, patients should be closely followed up in the first two years after starting treatment and continued long-term surveillance by clinical and imaging examination is warranted.

## Figures and Tables

**Table 1:** Summary of tumour classification systems AJCC 8th edition and BWH.

AJCC-8 Classification		BWH Classification	
**Primary tumour (T)**			
**T1**	Tumour < 2 cm in greatest dimension	**T1**	0 high-risk factors ^b^
**T2**	Tumour ≥ 2 cm and <4 cm in greatest dimension	**T2a**	1 high-risk factor
**T3**	Tumour ≥ 4 cm in greatest dimension or minor bone erosion or PNI or deep invasion ^a^	**T2b**	2–3 high-risk factors
**T4a**	Tumour with gross cortical bone/marrow invasion	**T3**	4 high-risk factors or bone invasion
**T4b**	Tumour with axial skeleton invasion including foraminal involvement and vertebral foramen involvement to the epidural space		
**Regional lymph nodes (N)**			
**Nx**	Regional lymph nodes cannot be assessed		
**N1**	Metastasis in a single ipsilateral lymph node ≤3 cm in greatest dimension and ENE (−)		
**N2a**	Metastasis in a single ipsilateral lymph node >3 cm and ≤6 cm in greatest dimension and ENE (−)		
**N2b**	Metastasis in multiple ipsilateral nodes all ≤6 cm in greatest dimension and ENE (−)		
**N2c**	Metastasis in bilateral or contralateral lymph node(s), all ≤6 cm in greatest dimension and ENE (−)		
**N3a**	Metastasis in a lymph node >6 cm in greatest dimension and ENE (−)		
**N3b**	Metastasis in any lymph node(s) and ENE (+)		
**Distant metastasis (M)**			
**M0**	No distant metastasis		
**M1**	Distant metastasis		

AJCC, American Joint Committee on Cancer; BWH, Brigham and Women’s Hospital; ENE, extranodal extension; PNI, perineural invasion. ^a^ Deep invasion defined as invasion beyond the subcutaneous fat or >6 mm (as measured from the granular layer of adjacent normal epidermis to the base of the tumour); perineural invasion for T3 classification is defined as tumour cells within the nerve sheath of a nerve lying deeper than the dermis or measuring 0.1 mm or larger in calibre or presenting with clinical or radiographic involvement of tumour named nerves without skull base invasion or transgression [19]. ^b^ Risk factors include tumour diameter 2 cm or larger, poorly differentiated histology, perineural invasion, and tumour invasion beyond the subcutaneous fat (excluding bone, which automatically upgrades to T3) [20].

**Table 2:** Staging based on AJCC TNM classification 8th edition for head and neck cSCC.

T	N	M	Stage
T1	N0	M0	Stage I
T2	N0	M0	Stage II
T3	N0, N1	M0	Stage III
T1	N1	M0	Stage III
T2	N1	M0	Stage III
T1–3	N2	M0	Stage IV
T1–4	N3	M0	Stage IV
T4	N0–3	M0	Stage IV
T1–4	N0–3	M1	Stage IV

AJCC, American Joint Committee on Cancer; M, distant metastasis; N, regional lymph nodes; T, primary tumour.

**Table 3:** Main characteristics of imaging modalities used in cSCC.

Imaging Modality	Optimal Use in cSCC	Advantages	Disadvantages	Sensitivity/Specificity for H&N Nodal Disease ^a^
CT	Bone or lymph node disease	Less expensive, more widely available, and faster image acquisition than MRI	Exposure to contrast dye and ionizing radiation	52%/93%
MRI	Perineural, CNS, deep soft tissue, BM, or lymph node disease	No exposure to ionizing radiation	Less widely available, longer acquisition time, more expensive than CT	65%/81%
US	Superficial lymph node disease and image-guided FNA	Least expensive, no exposure to contrast dye or ionizing radiation, rapid image acquisition, global accessibility	Operator and technique-dependent, limited visualization of deep structures	66%/78%
PET/CT	Distant metastases	Functional and anatomic information, distinguishes postoperative scar tissue from recurrence	Most expensive, lesions less than 10 mm are below resolution for FDG-PET	66%/87%

BM, bone marrow; CNS, central nervous system; cSCC, cutaneous squamous cell carcinoma; CT, computed tomography; FDG, fluorodeoxyglucose; FNA, fine needle aspiration; H&N, head and neck; MRI, magnetic resonance imaging; PET, positron emission tomography; US, ultrasonography. ^a^ Adapted from Liao et al., 2012 [39].

**Table 4:** Clinical and pathological features for risk stratification of cSCC.

	Low-Risk cSCC	High-Risk cSCC
**Clinical history and parameters**		
Location/size	Area L < 20 mm Area M < 10 mm	Area L ≥ 20 mm Area M ≥ 10 mm Area H
Borders	Well-defined	Poorly defined
Primary vs. recurrent	Primary	Recurrent
Immunosuppression	-	+
Prior RT or chronic inflammatory process	-	+
Rapidly growing tumour	-	+
Neurologic symptoms	-	+
**Pathology**		
Degree of differentiation	Well or moderately defined	Poorly defined
Subtypes		
Acantholytic (adenoid), adenosquamous, desmoplastic, or metaplastic (carcinosarcomatous) subtypes	-	+
Depth: thickness or level of invasion	≤6 mm, no invasion beyond subcutaneous fat	>6 mm or invasion of subcutaneous fat
Perineural, lymphatic, or vascular involvement	-	+

cSCC, cutaneous squamous cell carcinoma; RT, radiotherapy. Area H: “mask areas” of face (central face, eyelids, eyebrows, periorbital, nose, lips (cutaneous and vermilion), chin, mandible, preauricular and postauricular skin/sulci, temple, and ear), genitalia, hands, and feet. Area M: cheeks, forehead, scalp, neck, and pretibial. Area L: trunk and extremities (excluding hands, nail units, pretibial, ankles, and feet). Adapted from NCCN guidelines for SCC, 2018 [7].

**Table 5:** Suggested treatments with RT using external beam irradiation.

Type RT	Observations	Dose (Gy)	Sessions (n)	Times/wk	Fractionation
Definitive	Standard, GPS, size > 2 cm	64–66	32–33	5	conventional
	Neck, no surgery	70	35	5	conventional
	Size < 2 cm	50	20	5	hypofractionation
	Frail patients + inconvenience	50	15	5	hypofractionation
	Frail patients	36,75	7	5	hypofractionation
	Frail patients	35	5	3–5	hypofractionation
Adjuvant	Positive margins	66	33	5	conventional
	Negative margins	60	30	5	conventional
		50	20	5	hypofractionation
	Elective	50–54	25–27	5	conventional

GPS, good performance status; RT, radiotherapy; wk, week.

**Table 6:** Trials in loco-regional and/or metastatic unresectable disease with targeted therapies.

Drug	Phase (*N*)	Patient Characteristics	RR	DC	mDoR	mPFS	mOS	Ref.
Erlotinib	II (39)	PS 0–2 Median age 68 y	10% (no CR)	72%	7.2 mo	4.7 mo	13 mo	[64]
Gefitinib	II (40)	PS 0–2 Median age 67 y	16% (no CR)	51%	31.4 mo	3.8 mo	12.9 mo	[65]
Cetuximab	II (36)	PS 0–2 Strong/moderate EGFR expression Median age 79 y	28% (6% CR)	68%	6.8 mo	4.1 mo	NR	[66]
Panitumumab	II (16)	PS 0–2 Median age 68 y	31% (12% CR)	69%	6 mo	8 mo	11 mo	[67]

CR, complete response; DC, disease control; DoR, duration of response; mo, months; m, median; N, patient number; NR, not reached; OS, overall survival; PFS, progression-free survival; PS, performance status; RR, response rate; y, years.

**Table 7:** Selected ongoing clinical trials of immunotherapy in cSCC.

Immunotherapy	Treatment	Patients	NCT Code
Cemiplimab	Alone, pre-operative therapy, intralesional	Recurrent cSCC	NCT03889912
	Adjuvant therapy, after surgery and radiotherapy	High-risk cSCC	NCT03969004
	Alone or in combination with RP1	Advanced or metastatic cSCC	NCT04050436
	Alone	Unresectable locally recurrent and/or metastatic cSCC	NCT04242173
	Alone, neoadjuvant therapy	Stage II to IV cSCC	NCT04154943
Pembrolizumab	Alone	Recurrent/metastatic or locally advanced unresectable cSCC	NCT03284424
	Alone	Locally advanced or metastatic cSCC	NCT02964559
	Adjuvant therapy, after surgery and radiotherapy	High risk locally advanced cSCC	NCT03833167
	Combination with cetuximab	Recurrent/metastatic cSCC	NCT03082534
Nivolumab	Alone	Locally advanced/metastatic cSCC	NCT04204837
	Alone	Advanced cSCC	NCT03834233
	Alone or in combination with ipilimumab	Metastatic cSCC in immunosuppressed patients	NCT03816332

## Data Availability

The authors confirm that all data supporting the findings of this study are available within the article and its Supplementary Materials.

## Supplementary Materials

The following are available online at https://www.mdpi.com/article/10.3390/cancers14030629/s1, Table S1: Methods and results of the Delphi survey.
