# Supplementary material for: Update on Management Recommendations for Advanced Cutaneous Squamous Cell Carcinoma
Source: Cancers (Basel). 2022 Jan 27;14(3):629. doi: 10.3390/cancers14030629 (PMC8833756; doi:10.3390/cancers14030629)
Supplement: Supplementary file 1 [file cancers-14-00629-s001.zip › cancers-1515329-supplementary.pdf]

cancers

MDPI

Supplementary files

Update on Management Recommendations for Advanced Cutaneous Squamous Cell Carcinoma

Jesús García-Foncillas, Antonio Tejera-Vaquerizo, Onofre Sanmartín, Federico Rojo, Javier Mestre, Salvador Martín, Ignacio Azinovic and Ricard Mesía

Delphi survey

SUPPLEMENTARY METHODS

Study design and participants

A panel of experienced specialists were asked to answer a set of questions, developed by the scientific committee, in a two-round Delphi study [1]. The scientific committee was composed of eight specialists with extensive experience in the management of cutaneous squamous cell carcinoma. It included one specialist from pathological anatomy, three medical oncologists, one surgeon, two dermatologists, and one radiation oncologist.

Participants were selected based on their clinical experience in the management of cSCC at medical oncology, dermatology, pathological anatomy, and surgery units within the Spanish National Health System. All participants were contacted via e-mail, informed of the study characteristics, and asked for their consent to participate in it, prior to completion of the electronic questionnaire.

Questionnaire and analysis

Based on the literature search performed to address recommendations on the management of cSCC, the scientific committee proposed several questions to be included in the Delphi questionnaire. A total of 19 statements were grouped into four sections: prognosis (n = 2 statements), diagnosis (n = 2), treatment (n = 11), and follow-up (n = 4). In the first round, participants were requested to rate each statement on a 9-point Likert-scale to assess their agreement or disagreement with it (1 = totally disagree; 9 = totally agree) [2]. A second round was not needed since consensus was reached in all statements in the first round.

Statements were classified as appropriate when a median score of 7 or higher was recorded. If the median score was between 1 and 3, the statement was deemed inappropriate, while if it was within the 4 to 6 range, it was considered uncertain. The mean absolute deviation from the median (MAD-M) was used to measure the statistical dispersion. Consensus was based on the RAND Healthcare corporation and University of California at Los Angeles (RAND/UCLA) Appropriateness Method [3]. Data was analyzed using the R statistics package version 3.2.5.

SUPPLEMENTARY RESULTS

After the first round of the Delphi questionnaire, agreement on appropriateness was achieved in all the statements regarding the prognosis, diagnosis, treatment, and follow-up of cSCC (**Table S1**).

Prognosis

Panelists agreed that evidence and clinical practice support that high-risk prognostic factors for cSCC should include: tumour diameter (>20 mm); localization on temple/ear/lip area (if >10mm); thickness >6 mm or invasion beyond subcutaneous fat; poor grade of differentiation; desmoplasia; microscopic, symptomatic, or radiological PNI;

bone erosion; and immunosuppression. Likewise, a strong consensus was deemed on the features of regional disease that frequently considered prognostic factors for cSCC. Additionally, panelists proposed to include in-transit metastasis, pre-existing chronic ulcer/scar, the presence of vascular and/or neural invasion, immunocompromised status, and previous treatments.

Diagnosis

Participants agreed that the histopathological diagnosis of cSCC should include at least the following elements: histological grade; diameter and maximum tumour size; perineural/ lymphovascular invasion; complete removal; positive/negative margins; distance from the tumour to the lateral/deep margin; and budding. Besides, deep invasion of the tumour and the subtype of SCC were considered relevant. All panelists agreed on the appropriateness of the statement highlighting the need to identify clinically useful and validated molecular alterations to improve both prognosis and management in patients with metastatic cSCC.

Treatment

The choice of the most appropriate treatment depends on patient's functional status as well as stage, size, and location of the tumour. Panelists agreed that cemiplimab could be used in earlier phases of cSCC in selected cases for which no better treatment options are available, yet prospective clinical trials are needed for further implementation. The use of diagnostic imaging in SCC is recommended to assess the local extension of the tumour and its regional involvement. A consensus was reached regarding clinical evaluation and complementary studies of the regional nodes, which are recommended in high-risk cSCC patients. Treatment of cSCC should be performed by one or two surgical teams with enough experience in performing both the tumour exeresis and subsequent restoration. It is advisable to biopsy lesions suggestive of cSCC that are refractory to treatment to confirm diagnosis and assist in the identification of prognostic factors. Mohs micrographic surgery is the preferred technique for high-risk patients.

Follow-up

Panelists strongly agreed that patients should be trained, if possible, in self-examination of the scar, surrounding tissue and regional nodes to facilitate early diagnosis of relapses and lymphatic metastases. The frequency of follow-up visits should be adapted individually, depending on patient-specific underlying risk characteristics for cSCC, but also considering additional factors. Imaging examination should be performed every 3-6 months in the first 3 years and then based on individual symptoms and stage. However, the follow-up schedule should be adjusted for patients at very high risk for multiple primary tumours.

Table S1. Results of the Delphi survey.

	Statement	Median (MAD-M)	IQR	Max	Min	Result
	**Section 1: PROGNOSIS**					
1	Evidence and clinical practice support that high-risk prognostic factors for cSCC should include: tumor diameter (>20 mm); localization on temple/ear/lip area (if >10mm); thickness >6 mm or invasion beyond subcutaneous fat; poor grade of differentiation; desmoplasia; microscopic, symptomatic, or radiological PNI; bone erosion; and immunosuppression.	9 (0.4)	0.5	9	7	Appropriate

2	Features of the regional disease that are frequently considered prognostic factors for cSCC include: lymph nodes size, extracapsular extension, number of involved nodes, and involvement of parotid, neck nodes, or both.	9 (0.5)	1.0	9	7	Appropriate
	**Section 2: DIAGNOSIS**					
3	In patients with cSCC, the histopathological diagnosis should include at least the following elements: histological grade; diameter and maximum tumor size; perineural/ lymphovascular invasion; complete removal; positive/negative margins; distance from the tumor to the lateral/deep margin; and budding.	9 (0.6)	1.0	9	7	Appropriate
4	In patients with metastatic cSCC, there is a need to identify clinically useful and validated molecular alterations to improve both prognosis and management through tailored approaches.	9 (0.6)	1.0	9	7	Appropriate
	**Section 3: TREATMENT**					
5	Cemiplimab could be used in earlier phases of cSCC in selected cases for which no better treatment options are available, yet prospective clinical trials are needed for further implementation.	8 (1.0)	1.5	9	5	Appropriate
6	The indication of cemiplimab in patients with no other local treatment options would potentially move cetuximab to second line.	7 (1.1)	2.0	9	5	Appropriate
7	The use of diagnostic imaging (CT, MRI, US) in SCC is recommended to assess the local extension of the tumour and its regional involvement when there is suspicion.	9 (0.7)	1.0	9	6	Appropriate
8	Regional assessment is indicated in cSCC patients at high risk to identify lymphatic metastases.	8 (0.7)	1.0	9	6	Appropriate
9	Clinical evaluation and complementary studies of the regional nodes are recommended in high-risk cSCC patients.	9 (0.7)	1.0	9	5	Appropriate
10	Treatment of cSCC should be performed by one or two surgical teams with enough experience in performing both the tumour exeresis and subsequent restoration, thus ensuring the margins of resection are not conditioned.	9 (0.5)	0.5	9	7	Appropriate
11	It is advisable to biopsy lesions suggestive of cSCC that are refractory to treatment to confirm diagnosis and assist in the identification of prognostic factors.	8 (1.5)	2.5	9	3	Appropriate
12	The choice of the most appropriate treatment depends on patient's functional status as well as stage, size and location of the tumour.	8 (0.7)	1.0	9	5	Appropriate
13	Mohs micrographic surgery is the preferred technique for high-risk patients based on the high cure rates and low recurrence rates reported.	7 (1.1)	2.5	9	5	Appropriate
14	Safety margins should be: 4 mm in low-risk cSCC, 6 mm in moderate-risk cSCC, and 1 cm or Mohs surgery in high-risk cSCC.	8 (1.5)	4.0	9	5	Appropriate
15	Ultrasound/CT evaluation of regional T2b nodes is indicated because of their risk of metastasis.	8 (0.9)	2.0	9	5	Appropriate
	**Section 4: FOLLOW-UP**					

16	Patients should be trained, if possible, in self-examination of the scar, surrounding tissue and regional nodes to facilitate early diagnosis of relapses and lymphatic metastases.	8 (1.1)	2.0	9	5	Appropriate
17	The frequency of follow-up visits should be adapted individually, depending on patient-specific underlying risk characteristics for cSCC: low- vs. high-risk primary tumour, local vs. regional disease, presence of metastases and immunosuppression setting.	8 (1.0)	2.0	9	5	Appropriate
18	Imaging examination should be performed in patients with regional, locally advanced or metastatic cSCC every 3-6 months in the first 3 years and then based on individual symptoms and stage.	7 (0.8)	1.5	9	5	Appropriate
19	In patients at very high risk for multiple primary tumours, the follow-up schedule should be adjusted depending on the number and frequency of developing other tumours.	9 (1.0)	2.0	9	5	Appropriate

Abbreviations: IQR, interquartile range; MAD-M (mean absolute deviation from the median). Physicians rated their agreement with the statements using a nine-point Likert scale (one = totally disagree and nine = totally agree). **Notes:** Statements were classified as appropriate (median ranged from seven to nine), irrelevant (median ranged from four to six) or inappropriate (median ranged from one to three). Agreement was established when at least two thirds of the panel scored within any of the ranges (appropriate, irrelevant or inappropriate). Absence of consensus was considered when less than two thirds of the panel scored within the range containing the median, and 'controversy' when more than one third of individual scores were within the range opposite the one containing the median.

References

1. Diamond, I.R.; Grant, R.C.; Feldman, B.M.; Pencharz, P.B.; Ling, S.C.; Moore, A.M.; Wales, P.W. Defining consensus: A systematic review recommends methodologic criteria for reporting of Delphi studies. *J. Clin. Epidemiol.* **2014**, *67*, 401–409
2. Norman, G. Likert scales, levels of measurement and the "laws" of statistics. *Adv. Health Sci. Educ. Theory Pract.* **2010**, *15*, 625–632.
3. Fitch, K.; Bernstein, S.J.; Aguilar, M.D.; Burnanrd, B.; LaCalle, J.R.; Lazaro, P.; van het Loo, M.; McDonell, J.; Vader, J.; Kahan, J.P. *The Rand/UCLA Appropriateness Method User's Manual*; RAND Corporation: Santa Monica, CA, USA, 2001. Available online: https://www.rand.org/pubs/monograph_reports/MR1269.html (accessed on 3 March 2021).
